# Effects of exercise countermeasures on multisystem function in long duration spaceflight astronauts

**DOI:** 10.1038/s41526-023-00256-5

**Published:** 2023-02-03

**Authors:** Jessica M. Scott, Alan H. Feiveson, Kirk L. English, Elisabeth R. Spector, Jean D. Sibonga, E. Lichar Dillon, Lori Ploutz-Snyder, Meghan E. Everett

**Affiliations:** 1 Memorial Sloan Kettering Cancer Center, New York, NY USA; 2 Weill Cornell Medical College, New York, NY USA; 3 National Aeronautics and Space Administration (NASA), Houston, TX USA; 4 Milligan University, Milligan College, Elizabethton, TN USA; 5 KBR, Houston, TX USA; 6 University of Texas Medical Branch, Galveston, TX USA; 7 University of Michigan, Ann Arbor, MI USA

**Keywords:** Physiology, Medical research

## Abstract

Exercise training is a key countermeasure used to offset spaceflight-induced multisystem deconditioning. Here, we evaluated the effects of exercise countermeasures on multisystem function in a large cohort (*N* = 46) of astronauts on long-duration spaceflight missions. We found that during 178 ± 48 d of spaceflight, ~600 min/wk of aerobic and resistance exercise did not fully protect against multisystem deconditioning. However, substantial inter-individual heterogeneity in multisystem response was apparent with changes from pre to postflight ranging from −30% to +5%. We estimated that up to 17% of astronauts would experience performance-limiting deconditioning if current exercise countermeasures were used on future spaceflight missions. These findings support the need for refinement of current countermeasures, adjunct interventions, or enhanced requirements for preflight physiologic and functional capacity for the protection of astronaut health and performance during exploration missions to the moon and beyond.

## Introduction

For over 50 years International Space Agencies have continually refined countermeasures to protect astronaut health and performance from the multisystem physiological deconditioning that occurs during spaceflight^1^. Initially, exercise countermeasures consisted of elastic bands that provided little, if any, protection against spaceflight-induced deterioration in cardiorespiratory fitness and muscle size and strength^2,3^. Since these early missions, increasingly advanced exercise hardware was developed such that astronauts on International Space Station (ISS) missions now complete exercise training sessions using the Advanced Resistive Exercise Device (ARED), second generation treadmill (T2), and cycle ergometer with Vibration Isolation and Stabilization System (CEVIS).

Planned lunar surface and deep space exploration missions may last up to three years during which astronauts will be exposed to microgravity during transport and partial gravity during surface stays on the Moon or Mars. Prior studies evaluating the physiological effects of spaceflight were limited by the small number of astronauts (*n* < 30)^4^, the use of older exercise countermeasure devices that were restricted in speed and/or load^5^, the absence of assessment of countermeasures^6^, and/or the evaluation of effects on a single system^5,7–9^. There is therefore a significant need to evaluate the efficacy of current ISS countermeasures to determine whether modifications are needed for future human exploration missions. Here, we evaluated the effects of ISS exercise countermeasures on multisystem function, characterized heterogeneity in multisystem changes, and estimated the proportion of astronauts that would experience performance-limiting deconditioning on future missions.

## Results

### Overall approach and astronaut characteristics

All National Aeronautics and Space Administration (NASA), Canadian Space Agency (CSA), European Space Agency (ESA), and Japan Aerospace Exploration Agency (JAXA) astronauts assigned to ISS flight were eligible to participate in this investigation. Testing was performed during ISS Increments 26S-50S (April 2011 – September 2017). Forty-six astronauts (37 males, 9 females; age: 46.8 ± 6.1 years, height: 176 ± 7.1 cm, weight: 79.2 ± 9.9 kg [mean ± SD]) were assigned to missions of 178 ± 48 d. 10 (22%) astronauts had previously completed long-duration ISS missions. All astronauts performed the standard medically required physiologic tests assessing muscle strength, aerobic fitness, and bone health; a subset performed additional experimental tests of muscle strength and size, aerobic fitness, and bone health.

### Inflight exercise training and food systems during ISS missions

Inflight aerobic exercise was performed using the T2 (Supplemental Figure 1) and the CEVIS (Supplemental Fig. 2), and resistance exercise was performed with the ARED (Supplemental Fig. 3). Inflight exercise data are presented in Table 1. Median [interquartile range (IQR)] number of completed aerobic exercise sessions was 65 (47, 76) and 84 (59, 109) for CEVIS and T2, respectively. Inflight resistance exercise training load was 181 lbs/session (150, 223), 192 lb (156, 215), 239 lb (198, 293), 122 lb (122, 153) for squats, deadlift, calf raises, and bench press, respectively. Total exercise time (*i.e*., T2, CEVIS, and ARED) was ~600 min/wk per crew member (range: ~450 min/wk to 720 min/wk). Dietary intake during flight was recorded using multiple techniques, as this has changed over time on ISS. On average, astronauts consumed a total of 2296 ± 449 kcal/day, corresponding to 29 ± 5 kcal/kg/day (Supplemental Table 1).

**Table 1:** Inflight Aerobic and Resistance Exercise Training.

	Aerobic Exercise		Resistance Exercise			
	CEVIS	T2	Deadlift	Heel Raise	Squat	Bench Press
Sessions, number	65 (47, 76)	84 (59, 109)	121 (82, 146)	85 (65, 105)	117 (78, 148)	
^a^Exercise time/session, mins	26 (23, 29)	27 (23, 29)	N/A	N/A	N/A	N/A
Heart Rate, beats/min						
Average	135 (128, 141)	131 (121, 137)	N/A	N/A	N/A	N/A
Peak	158 (150, 163)	153 (144, 162)	N/A	N/A	N/A	N/A
% time in heart rate zone						
Above 70% peak heart rate	76 (65, 92)	69 (54, 81)	N/A	N/A	N/A	N/A
Above 90% peak heart rate	13 (6, 27)	11 (4, 24)	N/A	N/A	N/A	N/A
Speed, rpm (CEVIS) or mph (T2)						
All	78 (71, 97)	7 (6, 7)	N/A	N/A	N/A	N/A
Peak	92 (88, 97)	8 (7, 9)	N/A	N/A	N/A	N/A
Load, W (CEVIS) or lb (T2)						
All	137 (123, 153)	117 (107, 127)	192 (156, 215)	239 (198, 293)	181 (150, 223)	122 (98, 153)
Peak	191 (173, 223)	N/A	220 (180, 248)	249 (202, 306)	229 (194, 270)	131 (102, 160)
Repetitions, number	N/A	N/A	230 (177, 313)	207 (131, 256)	189 (135, 233)	44 (38, 81)
Load volume	5013 (3864, 5763)	2909 (2531, 3279)	38,300 (28,770, 61,681)	45,855 (32,410, 67,364)	31,659 (22,745, 46,150)	4928 (4601, 10,311)

*N/A* not applicable, *IQR* interquartile range, *rpm* revolutions per minute, *mph* miles per hour, *W* Watts, *lb* pounds.

Data presented as median, IQR

^a^Exercise time does not include warm up or cool down time.

Load volume, exercise time x all load (aerobic) or reps x all load (resistance).

### Change in multisystem function

**Fig. 1:**
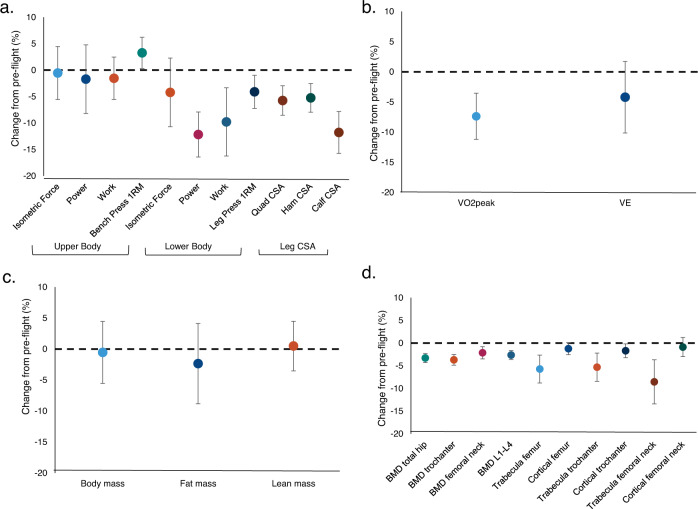
Estimates of mean percent change. (**a**) muscle strength and size; (**b**) cardiorespiratory fitness; (**c**) body composition, and (**d**) bone mineral density and bone content. Data are mean and 95% confidence interval (CI).

A total of 27 performance and/or physiological endpoints were collected across four systems (muscle, cardiorespiratory, bone, and body composition) during preflight and postflight ground-based testing (sample size for each endpoint varies due to astronaut testing schedules; Supplemental Table 2). For each of these four systems, descriptive summaries are shown respectively in Supplemental Tables 3**–**6. Estimates of percent change indicate mean lower leg muscle cross-sectional area and strength were significantly decreased (*p* < 0.05); but there was no evidence of a similar change in mean upper body muscle strength (Fig. 1a). On average, cardiorespiratory fitness (VO_2_peak) declined by 7.4% ± 2.0% from preflight to postflight (Fig. 1b). Means of total body mass, lean mass, and fat mass were virtually unchanged postflight (Fig. 1c). Changes in mean bone mineral density (BMD) were moderate, ranging from −2.1% ± 0.7% to −3.7% ± 0.6%; however much greater declines were observed for bone content in the trabecular regions [(−5.3% ± 1.6% to −8.5% ± 2.5%); (Fig. 1d)].

### Variability in multisystem responses

**Fig. 2:**
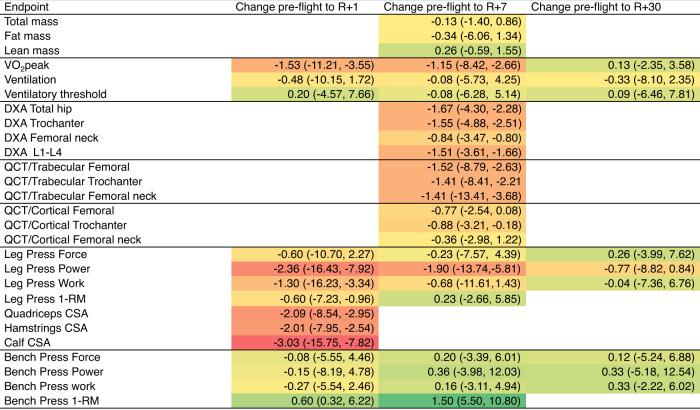
Estimated effect sizes of change across multisystem function. Effect size estimates are color-coded to reflect their signs and magnitudes with darker colors reflecting larger losses or gains. Abbreviations: QCT Quantitative Computed Tomography, DXA Dual Energy X-ray Absorptiometry; RM repetition maximum, CSA cross-sectional area, VO_2_peak peak oxygen consumption. Data are mean estimated effect size and 95% confidence interval (CI).

There was substantial inter-individual heterogeneity in response across all endpoints. Supplemental Figure 4 outlines exemplar individual responses for quadriceps size (Supplemental Figure 4A), VO_2_peak (Supplemental Figure 4B), and bone content in trabecula femur (Supplemental Figure 4C). Given the observed variability in pre to postflight change between systems, we next estimated change effect sizes to quantify signal-to-noise ratios independent of sample size, where “signal” is the mean change and “noise” is the within-subject standard deviation of repeated preflight measurements. Estimated effect sizes obtained after fitting mixed-model regression show negative effect sizes for 23 endpoints with large losses (−1.0 or lower) for cardiorespiratory fitness, lower-body muscle size and strength, and all bone health endpoints, whereas smaller effect sizes were observed for body composition and upper body muscle strength endpoints (Fig. 2). Of the 11 endpoints that were serially evaluated postflight, the means of 9 were at or near preflight values by postflight 2 (~7 days postflight); however, mean cardiorespiratory fitness and leg press power remained below preflight values at postflight 3 (~30 days postflight) (Fig. 2).

### Factors associated with multisystem responses

**Fig. 3:**
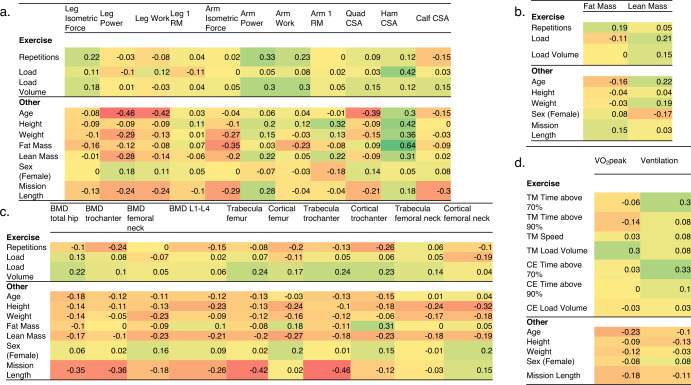
Association between baseline characteristics and inflight countermeasures with change in. (**a**) muscle, (**b**) body composition, (**c**) bone health and (**d**) cardiorespiratory fitness. Correlations are color-coded to reflect magnitude with darker colors reflecting higher correlation. Abbreviations: RM repetition maximum, CSA cross-sectional area, BMD bone mineral density, TM treadmill, CE cycle ergometer, VO_2_peak peak oxygen consumption, time above 70% and 90%, time in heart rate zone above 70% and 90% of peak heart rate, respectively. Data are rank-based Somers’ D.

We used rank-based Somers’ D^10^ to evaluate the association between in-flight exercise and other characteristics with change in muscle (Fig. 3A), body composition (Fig. 3B), bone health (Fig. 3C), and cardiorespiratory fitness (Fig. 3D) endpoints. This integrated matrix facilitates viewing specific correlations and general trends across systems. In general, longer mission length was associated with greater loss of bone content, lower body muscle strength, and VO_2_peak. Relatively large negative correlations were found between age and change in leg power (D = -0.46), leg work (D = −0.42), and VO_2_peak (D = −0.23). In contrast, higher resistance exercise volume load was associated with increased lower leg muscle strength and size, bone health, and lean mass, while treadmill volume was inversely associated with VO_2_peak. Change in bone health was correlated with losses in body weight, lean mass, and fat mass, indicating that both quantity and quality of exercise are important in maintaining bone health.

### Multisystem function and programmatic risk

Primary goals of NASA are to protect astronaut health and performance and to safely and efficiently complete mission tasks such as extravehicular activity (EVA) and vehicle egress after landing back on Earth or on a partial gravity surface. Mission performance is associated with specific absolute and quantifiable physiologic and functional capabilities^4,11,12^. We therefore quantified the risk of reduced performance to estimate programmatic risk on future exploration missions to provide operationally critical information to NASA program leaders. Clinical thresholds for reductions in health and performance are likely not directly relevant to the astronaut task performance criteria because of the physical and cognitive demand to perform tasks with extremely high mortality risk and small error margin. Consequently, in this paper we used previous spaceflight analog literature to define thresholds. Specifically, we defined high risk as a 20% or greater reduction in an endpoint because that threshold was associated with significant performance decrements in a ground-based analog study that evaluated simulated EVA and egress task performance^4,11,12^. We used mixed-model regression to estimate *P*_*20*_, the proportion of astronauts that would be expected to have a 20% or greater loss at the first postflight session. As outlined in Table 2, *P*_*20*_ was highest for lower-body work [*P*_*20*_ = 17%, 95% confidence interval (CI): 7%, 36%], lower-body power (*P*_*20*_ = 14%, 95% CI: 6%, 33%), calf muscle size (*P*_*20*_ = 11%, 95% CI: 3%, 31%), and cardiorespiratory fitness (*P*_*20*_ = 7%, 95% CI: 2%, 22%). *P*_*20*_ was negligible for all body composition and bone endpoints except for the trabecular content of the femoral neck (*P*_*20*_ = 15%, 95% CI: 6%, 33%).

**Table 2:** Proportion of astronauts that would be expected to have a 20% or greater loss at the first postflight session.

Endpoint	*P*_*20*_	95% Confidence interval	
Muscle strength, power, and size			
Leg Press Work	17.3	7.5	36.0
Leg Press Power	14.5	5.8	32.7
Bench Press Power	12.7	4.8	30.1
Calf CSA	10.9	3.2	30.9
Leg Press Force	8.8	2.8	24.9
Bench Press Work	3.6	0.9	14.3
Bench Press Force	2.2	0.4	11.2
Leg Press 1RM	2.0	0.6	6.0
Bench Press 1RM	0.5	0.1	2.6
Quadricep CSA	0.1	0.0	3.8
Hamstring CSA	0.0	0.0	2.1
Cardiorespiratory fitness			
Peak Workload	12.7	4.4	32.3
VO_2_peak	7.3	2.3	22.2
Ventilation	6.6	1.9	21.8
Ventilatory threshold	0.0	0.0	2.1
Body composition			
Fat mass	4.0	1.5	9.9
BMD trochanter	0.0	0.0	0.0
BMD femoral neck	0.0	0.0	0.0
BMD total hip	0.0	0.0	0.0
BMD L1-L4	0.0	0.0	0.0
Body mass	0.0	0.0	0.0
Lean mass	0.0	0.0	0.0
Bone Volume			
Trabecula Femoral neck	14.7	5.8	32.7
Trabecula Femur	2.2	0.5	9.4
Trabecula Trochanter	1.8	0.4	8.6
Cortical femoral neck	0.0	0.0	0.0
Cortical trochanter	0.0	0.0	0.0
Cortical femur	0.0	0.0	0.0

*RM* repetition maximum, *CSA* cross sectional area, *VO2peak* peak oxygen consumption, *BMD* bone mineral density

Values are %, 95% confidence interval.

## Discussion

Here, we provide a comprehensive report of physiological adaptations to spaceflight with contemporary exercise countermeasures. In addition to demonstrating the exercise interventions were not fully protective against spaceflight-related multisystem declines, we estimated that up to 17% of astronauts on future missions would have 20% or greater loss in one of more of lower body muscle performance, bone health, and cardiorespiratory fitness. It is noteworthy that there were declines in almost all endpoints, suggesting that the cumulative multisystem decrements could result in a significant impact in the ability to perform physically demanding mission tasks.

Spaceflight-induced multisystem deconditioning was a significant concern observed after even short duration (~14 day) Mercury, Gemini, and Apollo missions^13^. Exercise was selected as a mandatory inflight intervention on all missions given its efficacy to improve multisystem capacity. Standard-of-care exercise on earlier ISS missions (2001-2009; mission length: 91 to 215 days) consisted of combined aerobic and strength training implemented using a first-generation treadmill with vibration isolation and stabilization (TVIS), CEVIS, and the interim resistive exercise device (iRED). The treadmill and iRED were, however, limited in speed (max: 11.3 km/h) and load (max: 136 kg)^14^, and exercise prescriptions therefore primarily consisted of high volume (~110 min/day), moderate intensity (55%-75% of VO_2_peak or repetition maximum) exercise^15^. Intriguingly, even with high exercise volumes, bone mineral density losses^9^, muscle atrophy^15^, and decrements in cardiorespiratory fitness^16^ were apparent. In 2009, in response to frequent TVIS and iRED hardware failures and anomalies, the ISS exercise hardware was upgraded to the T2 and the ARED to allow for higher speeds (max: 19.3 km/h) and loads (max: 272 kg). Our group recently reported that incorporation of high-intensity/lower volume exercise prescription in 12 astronauts reduced decrements in bone mineral density, muscle strength and endurance, and cardiorespiratory fitness after long-duration spaceflight^4^ relative to astronauts who exercised with the iRED and TVIS^7^. These findings, together with our current results in a larger cohort of astronauts, support the notion that current ISS exercise countermeasures provide improved protection of musculoskeletal and cardiorespiratory endpoints during long-duration spaceflight relative to previous countermeasures.

Nevertheless, the results here indicate that current exercise countermeasures appear insufficient to maintain preflight physiological and functional status and that additional optimization may be necessary to fully offset spaceflight-induced decline. During future missions, astronauts will likely be exposed to prolonged periods of microgravity and then exposed to Lunar gravity. It is not known whether the transition from prolonged periods in microgravity to Lunar gravity will constitute significant health and safety risks; however, based on findings from Apollo missions it is likely that astronauts will experience orthostatic intolerance, balance problems, and spatial orientation challenges^17^. Future exploration missions to the Moon or Mars will also require physiologically demanding tasks such as constructing habitats and operating geologic equipment^18^. Finally, these missions may also include return (splashdown) in the ocean, where astronauts may be required to perform physiologically demanding egress tasks unaided^19^. Thus, additional countermeasures may be required to offset spaceflight-induced deconditioning. For instance, during 70 days of bed rest (a spaceflight analog), exercise and nutrition countermeasures coupled with low-dose testosterone were protective against decrements in metabolic health^20^ and muscle^21^, but not against cardiac, or bone changes^22^. Recapitulation of ground-based loading cycles of daily activities during 84 days of bed rest attenuated, but did not eliminate, the decline in several musculoskeletal and cardiovascular health parameters^23^. Nevertheless, findings from systematic reviews indicate that nutritional countermeasures could ameliorate musculoskeletal and cardiopulmonary deconditioning^24^, while findings from bed rest studies indicate that including plyometric exercise (hopping and whole body vibration) may be adjunct options to mitigate musculoskeletal loss on exploration missions where resources are limited^25^. Finally, lower body negative pressure (LBNP) coupled with exercise could offset the spaceflight-induced headward shift in vascular and cerebrospinal fluid and mitigate declines in cardiorespiratory fitness^26^. To this end, Lee and colleagues^27,28^ demonstrated that LBNP and exercise maintained cardiorespiratory fitness during 30 days of bed rest.

Two additional points are noteworthy. First, the large collection of correlational data in Fig. 3 provides several intriguing conceptual views. Sex was not associated with any meaningful correlations suggesting that female astronauts are not at a disadvantage with respect to response to exercise countermeasures. However, age and mission length were important predictors, inversely associated with bone content, lower body muscle strength, and VO_2_peak. As evidenced by inverse correlations with mission length, many bone endpoints were vulnerable to increasing mission duration. Resistance and treadmill volume loads were the key countermeasure factors associated with improved strength, bone, body composition, and cardiorespiratory endpoints. These findings are important for the design of exercise devices and prescriptions for longer-duration exploration missions that require mid-mission performance in partial gravity environments, and underscore that many individual characteristics, as well as spaceflight factors beyond those characterized in our study, likely influence physiologic responses.

Second, the tools employed here provide an evidence-based method to evaluate the likelihood that astronauts will maintain threshold performance levels. We found the proportion of astronauts that could have a 20% or greater loss at the first postflight session was highest for lower body muscle size and strength endpoints. These findings, together with the inverse association between age and change lower body endpoints, suggest additional countermeasures targeting the lower body may be needed for older astronauts. Whether adjunct interventions could mitigate spaceflight-related changes in lower body muscle strength and size is not known. However, interventions such as protein supplementation and anti-inflammatory drugs could synergize with exercise training to offset the blunted anabolic response to exercise training in older individuals^29^. ISS EVAs are long-duration activities (up to 8 hours) and require a high level of cognitive effort, but they are relatively low physical intensity (~30% of maximal effort) and infrequently performed (~3 EVAs per 6-month mission). Oxygen utilization is monitored during all ISS EVAs from a safety perspective and the overall EVA intensity is dependent on the crewmember and the specific tasks comprising the EVA^30^. In comparison, partial gravity EVAs on the lunar surface not only will be more frequent (up to 3 to 4/week, and up to 24 total hrs per week) and performed on unknown and irregular terrain, but also will require new unrehearsed tasks with complex logistics and a higher level of physical and cognitive demand for some tasks (e.g., ambulation, habitat construction, geological sampling). Collectively, the findings herein can be used to understand task performance expectations, to select feasible and acceptable tasks for crew to perform, and to identify areas where additional technology or hardware is needed to assist with task performance.

### Perspectives

These findings highlight the need to better personalize countermeasures to target the endpoint of interest for each individual astronaut. To this end, several important research gaps should be addressed to optimize astronaut health, safety, and performance on long-duration missions. For example, the stratification of astronauts into homogeneous subgroups based on preflight and inflight characteristics should be performed to investigate whether targeted exercise prescriptions could improve individual responses^31^. Additional research evaluating multimodal exercise, nutrition, and other adjunct interventions are also needed. Finally, model systems such as human induced pluripotent stem cells, organoid, and organ-on-a-chip technologies should be leveraged to evaluate whether an astronaut’s own cells could allow for the development of personalized countermeasures prior to spaceflight, or modification of countermeasures during exploration mission^32^.

### Limitations

Our study limitations require consideration. First, this study represents a large cohort of astronauts on long-duration spaceflight missions; however, relative to ground-based trials this represents a relatively low number of participants. Second, because exercise is a mandatory intervention for all astronauts on ISS missions, the effects of exercise on multisystem function during spaceflight relative to a non-exercise control are not known, which may impact quality of evidence of human exercise training studies during spaceflight^33^. Although findings from ground-based studies using spaceflight analogs such as bed rest indicate that exercise mitigates a substantial amount of deconditioning^22^, there was considerable variability in the actual exercise performed with respect to the standard exercise prescription parameters of intensity, duration and frequency. Third, although we included numerous endpoints spanning multiple systems, standard measures did not include endpoints related to recently identified health concerns such as Spaceflight-Associated Neuro-Ocular Syndrome (SANS)^34^. Updated standard measures for ISS astronauts, however, include a breadth of additional core measurements related to cardiovascular, immunology, microbiology, and biochemistry. Additional research is needed to evaluate the effects of countermeasures on systems not evaluated in the present study. Fourth, countermeasures consisted of exercise on three different devices designed for use on the ISS. The feasibility and efficacy of exercise on exploration class exercise hardware, such as flywheel devices (currently planned for early Artemis missions) could differ^22^. Finally, other uncontrolled confounders such as diet composition, pharmacological use, and degree of radiation exposure could also contribute to the observed heterogeneity in physiological changes.

## Conclusions

In summary, we found that ~600 min/wk of aerobic and resistance exercise during International Space Station missions was not fully protective against multisystem deconditioning in the overall astronaut cohort. Near-future exploration class missions will not have an ISS-like suite of exercise hardware. One of the most notable differences is that no treadmill is planned for the initial phase of Artemis missions and the resistance exercise load quality may not be comparable to the ISS ARED. Exploration upmass, power, and volume limitations combined with the requirements for astronauts to perform more physically and cognitively demanding exploration tasks with increased autonomy (less ground-based support) highlight the necessity to develop integrated and optimized countermeasures targeted at protecting human performance. Our findings provide important information regarding countermeasures for spaceflight and suggest multimodal interventions will be required to optimize astronaut health, safety, and performance on future exploration missions to the Moon and Mars.

## Methods

### Overview of research design

All National Aeronautics and Space Administration (NASA), Canadian Space Agency (CSA), European Space Agency (ESA), and Japan Aerospace Exploration Agency (JAXA) astronauts assigned to ISS flight were eligible to participate in this investigation. This study was approved by the Institutional Review Board at NASA Johnson Space Center (JSC, Houston, TX), the Japan Aerospace Exploration Agency (JAXA) Institutional Review Board, the European Space Agency (ESA) Medical Board, and the Human Research Multilateral Review Board. All astronauts completed standard preflight medical screening, received clearance from flight surgeons, and provided written informed consent before participating in the study. All astronauts included in this paper performed the standard medically required physiologic tests involving muscle strength, aerobic fitness, and bone health; a subset performed additional experimental tests of muscle strength and size, aerobic fitness, and bone health. Inflight exercise and nutrition data were collected throughout the astronauts’ missions. Supplemental Figs. 1–3 are courtesy of NASA (https://www.nasa.gov/multimedia/guidelines/index.html) and the authors affirm that human research participants provided informed consent for publication of the images in NASA image gallery.

### Participants and facilities

Testing for this study was performed during ISS Increments 26S-50S (April 2011 – September 2017). 46 astronauts (37 males, 9 females; age: 46.8 ± 6.1 y, height: 176 ± 7.1 cm, weight: 79.2 ± 9.9 kg [mean ± SD]) were assigned to missions of 178 ± 48 d. This study was approved by the Institutional Review Board at NASA Johnson Space Center (JSC, Houston, TX), the Japan Aerospace Exploration Agency (JAXA) Institutional Review Board, the European Space Agency (ESA) Medical Board, and the Human Research Multilateral Review Board; all subjects provided written informed consent before participating in the study. All astronauts completed standard preflight medical screening and received clearance from their flight surgeons before participating in the tests included in this study. We acknowledge that some astronauts included in this study were also participants in other studies^4^. The goal of this paper however is to report multisystem adaptations to spaceflight from a large cohort of astronauts, and as such we included all available astronaut health data.

### Countermeasures

Inflight aerobic exercise was performed using the second-generation treadmill (T2) and the Cycle Ergometer with Vibration Isolation System (CEVIS), and resistance exercise was performed with the ARED^35^. Resistance exercise was prescribed 3-6 d/wk and aerobic exercise was prescribed 5-6 days per week. T2 was modified from a commercial Woodway Path treadmill (Woodway, Waukesha, WI) to support walking and running exercise between 2.4 and 19.3 km·h^-1^. The user is loaded via a shoulder and waist harness which is attached to bungee cords and terminally, the treadmill deck surface. CEVIS operates similarly to a standard cycle ergometer providing workloads between 25 and 350 W at pedal speeds from 30-120 revolutions per minute. Crewmembers wore cycling shoes that snapped into the pedals and strapped themselves with a belt to the CEVIS frame or used the frame handles to remain appropriately positioned on the cycle. ARED simulates free weights with a constant load of 11–272 kg provided by vacuum cylinders and an inertial load effected by flywheels placed in the load path; both barbell and cable exercises can be performed^36^.

Generally, aerobic exercise was 30-60 mins in duration and either prescribed as continuous steady exercise or in intervals. Resistance exercise prescriptions were approximately 60 min in duration and included upper and lower body exercise with the core group of exercises, including squats, deadlift, heel raise, bench press, overhead press, and upright rows. The program typically consists of 1.5–2.0 h per day total of aerobic and resistance exercise, each performed 6 d per week. Although 2.5 h are scheduled for daily exercise on the ISS^14^, typically, exercise time was divided into 30-45 min of aerobic training and 60–75 min of resistance training with hardware configuration and post-exercise hygiene comprising the remainder of total allotted time. Aerobic training consisted of interval or continuous steady-state exercise on either CEVIS or T2. CEVIS protocols were developed using the preflight VO_2_peak test with prescribed work rates (W) between 70-100% VO_2_peak. ASCRs adjusted the protocols throughout the mission based on individual performance during training sessions and crew feedback. T2 protocols were based on preflight training and prescribed at 70–100% HR_max_. For most crewmembers, external (harness/bungee) loading began at 60% bodyweight (static load measured when standing stationary on the treadmill belt) and increased as tolerated throughout the mission. Resistance training followed a 9-day periodized program with linear progression of loads and undulating volume across two 12-week mesocycles. After a two-week acclimatization period, loads were set at 70% of the repetition-maximum (RM) prescribed for that session (e.g., for a 4 ×6 repetition session, loads in Week 3 were 70% of 6-RM) with loading intensity increasing 5% each week. Strength increases over the first mesocycle allowed most crewmembers to reach intensities of 110-120% of their early mission repetition-maximums by Week 12. For the second mesocycle, loads were reduced to 70% of the crewmember’s new repetition-maximum and the progression of the first mesocycle was repeated. A variation of squat, deadlift, and heel raises were each prescribed daily for control subjects followed by rotating exercises focusing on upper body and stability musculature.

Aerobic exercise endpoints were CEVIS and T2 average session duration and average HR (b·min^−1^ and % maximum) for 30 s, 2 min, and 4 min intervals, and continuous sessions. Session durations and heart rate parameters were calculated for the periods of “active” exercise time on the cycle ergometer or treadmill, i.e., excluding warmup and cooldown periods at the beginning and end of each session, and containing only the interval/continuous exercise period and the time between intervals. The %maximum HR parameter was calculated as the average HR of the individual exercise sessions for that crew member, divided by the crew member’s HR_max_ (determined pre-flight as part of Peak Aerobic performance testing), then multiplied by 100%. For resistance exercise, total volume was calculated for each subject for the categories of squat, heel raise, and deadlift exercises, then normalized to mission duration (total volume/mission duration in days). Warmup exercises were not included in the data set. The 3 exercise categories included the following variations: “squat”: back squat, single leg squat, sumo squat; “heel raise”: heel raise and single leg heel raise; “deadlift”: deadlift, Romanian deadlift, and sumo deadlift, and bench press. For each exercise category, total volume was calculated for each subject by summing the volume (load x reps) for across the entire mission. In addition, average load (kg), average relative load (kg·kg bodyweight^-1^), average repetitions per session, and average repetitions per week were calculated for each subject for the 3 exercise categories. Aerobic and resistance exercise training variables were recorded and are presented descriptively. Aerobic time spent exercising was based on the start and end times of the main set of exercise and did not include warm-up and cool-down periods. Start and stop time stamps are part of the exercise data stream. For resistance exercise, exercise time was based simply on the start and stop time. Total exercise time was estimated including warm-up and cool-down times.

11 crewmembers used a weekly Food Frequency Questionnaire^37^. 12 crewmembers used an excel spreadsheet to log all intake, and more recently 20 crewmembers used an iPad App, the ISS Food Intake Tracker (ISS FIT). Nutrient intake data were determined using Nutrition Data System for Research software versions 2007, 2010, 2012, and 2014, developed by the Nutrition Coordinating Center, University of Minnesota.

### Endpoints

Most of the astronauts (*n* = 38) performed the bench press 1-repetition maximum and leg press 1-repetition maximum 60 to 90 days before flight, 5 to 7 days after landing, and once more 30 days after landing as previously described^38^. Briefly, to obtain a 1 repetition maximum for leg press, crewmembers completed a warm-up at ~50% load for 10 repetitions, the load was increased 15–20% each set with decreasing repetitions until the subject could only complete 1 repetition at which point the load was increased 5–10% until failure. Participants rested 3–5 mins between sets. To obtain a 1 repetition maximum for bench press crewmembers completed a warm-up at ~30% load for 10 repetitions, the load was increased 10-20% each set with decreasing repetitions until the subject could only complete 1 repetition at which point the load was increased 5-10% until failure.

A subset of the astronauts (*n* = 17) completed additional tests of upper and lower body muscle strength and performance 60 to 90 days before flight and up to 39 days after landing, categorized into 3 postflight phases: (Post1: R + 1, R + 2; Post2: R + 6 to R + 9, Post3: R + 25 to R + 39). Lower body muscle performance was determined before and after spaceflight using a leg press and bench press test battery recently developed in our laboratory^39^. Modified and instrumented leg press and bench press stations were used to assess isometric strength and dynamic power as previously described^40^. To measure upper and lower body isometric strength, subjects performed 3 maximal efforts for 5 s each with 30 s of rest between each effort. To assess upper and lower body dynamic power and work capacity, subjects performed 21 consecutive ballistic, concentric-only bench press and bilateral leg press actions with the load fixed at 30% (bench press) and 40% (leg press) of the measured maximal isometric force (MIF), which has previously been shown to elicit maximal power output^39^. A magnetic brake (Fitness Technology) was used to catch the weight as soon as the sled reached its peak height so that no eccentric muscle actions were performed. Power and total work were calculated^40^.

Cross-sectional area (CSA) of the lower leg muscles was obtained from MRI scans pre and postflight on 12 astronauts. Images were acquired from the level of the ankle mortise to the iliac crest. The methods and reliability of this technique have been previously reported by our laboratory^41^. Muscle cross-sectional area was manually traced using Image-J (National Institutes of Health, Bethesda, MD, USA, version 1.42)^42^.

Cardiorespiratory fitness was evaluated during upright peak cycle ergometry tests (Lode Excalibur Sport; Lode B.V., Groningen, the Netherlands) performed once or twice before launch (between L-90 d and L-28), and between 2 and 4 days after landing. The protocol consisted of a 3-minute warm-up at 50 W, followed by 1-minute stepwise increments of 25 W to volitional fatigue. Heart rate (HR) and heart rhythm were monitored continuously (GE CASE, GE Healthcare, Chicago, IL). Ventilation and expired gas fractions (F_E_O_2_ and F_E_CO_2_) were measured continuously using the Portable Pulmonary Function System (PPFS) as previously described^16^. VO_2_peak was defined as the highest 30-s average and was confirmed by the attainment of at least two of three criteria: 1) respiratory exchange ratio of > 1.09; 2) heart rate >90% of age-predicted maximum; 3) a plateau in VO_2_ (an increase of < 150 mL · min^−1^) from the previous stage. Ventilatory threshold was defined as the point at which VCO_2_ began to increase disproportionate to VO_2_ and V_E_/VO_2_ increased with no concomitant increase in V_E_/VCO_2_^43^.

Dual Energy X-ray Absorptiometry (DXA) scans were obtained using a single densitometer (Hologic Discovery; Hologic Inc., Waltham, MA, USA). Two bone densitometry technologists, certified by the International Society for Clinical Densitometry (ISCD), performed and analyzed the scans. For a given crewmember, a single technologist performed both the pre and postflight scans. Scans were performed at approximately 90 days preflight (L-90) and again 1-2 weeks after landing (R + 7). At each test session, the following fan-beam DXA scans were performed: left and right hip, lumbar spine, whole body, and left heel. Scans were performed and analyzed according to standard procedures recommended by the manufacturer, except for hip and heel scans. As reported for previous spaceflight and bed rest studies^44,45^, the global region of interest box for the hip was positioned manually, with the lateral margin placed adjacent to the lateral cortex of the greater trochanter and the distal border placed a set number of lines from the lesser trochanter’s distal margin. Heel scans were obtained using the forearm scan mode, with the subject seated on the scanner and the foot restrained in a lateral position within a custom jig. In addition to areal bone mineral density (BMD, g•cm^2^) obtained from the scans listed above, whole body and regional lean mass (fat-free, bone-free mass) and fat mass were determined from the whole body scans using standard Hologic analysis software. The BMD precision values (Least Significant Change, 95% confidence limit) for the scanning laboratory were as follows: total hip, 2.1%; trochanter, 3.0%; femur neck, 3.9%; lumbar spine, 2.3%; heel, 2.5%; and whole body, 2.8%. Precision (Least Significant Change, 95% confidence limit) of soft tissue values from the whole body scans were: whole body lean mass, 2.5% and whole body fat mass, 5.9%. Calibration of the Hologic densitometer was verified by regular scanning of a calibration phantom (at least weekly as well as on the day of subject testing), with scans analyzed using the manufacturer’s automated software.

Pre- and postflight CT scans were performed at a local hospital radiology center, using a single scanner (General Electric Advantage QXi) for all subjects. A single helical CT scan at each test session was used to image both the left and right hips (2.5-mm sections at 80 Kvp, 2880 mA), with a calcium hydroxyapatite phantom placed under the subjects’ hips during the scan as a reference standard. CT images were transferred to a computer workstation and processed to extract measures of volumetric BMD (vBMD) using analysis techniques described previously^46^. Processing included a step to calibrate the CT images from the native scanner Hounsfield Units to equivalent concentration (g/cm^3^) of calcium hydroxyapatite (HA) and determination of trabecular, cortical, and integral regions of interest for each of the left and right proximal femurs. For each region, Bone Mineral Content (BMC, g), and bone volume (cm^3^) were calculated, and these were then used to calculate vBMD (BMC/volume, g/cm^3^). The regions of interest included volumes of trabecular, cortical, and integral (trabecular + cortical) bone in the femoral neck in a region encompassing the greater and lesser trochanters and in an overall region comprising both the femoral neck and trochanter. The femoral neck was subtracted from the overall proximal femoral region to compute the region of the trochanter. Trabecular bone regions were determined by eroding the integral bone regions to produce regions of the same shape but fully contained within the medullary volumes. A threshold of 0.35 g/cm^3^ was used to define regions of cortical bone, i.e., voxels falling outside the trabecular regions but within the corresponding integral region.

### Statistical Methods

For each of the physiological and performance endpoints (Supplemental Table 2) mixed-model linear regression was used to estimate the mean response at each time point. These models work well to adjust for random data dropout, as is pervasive in this observational study. To compensate for non-normality of residuals, bootstrapping (200 samples) was used to obtain improved estimates of the standard-error matrix. For each postflight session (*k*), the percent change in the mean was estimated by $PCT_k = 100 \times \frac{{\hat \mu _k - \hat \mu _0}}{{\hat \mu _0}}$ where $\hat \mu _k$ is the estimated mean at the *k-*th postflight session, and $\hat \mu _0$is the estimate of the preflight mean. In addition, we estimated the effect size at each postflight session by $ES_k = 100 \times \frac{{\hat \mu _k - \hat \mu _0}}{{\hat \sigma }}$, where $\hat \sigma$ is the estimated within-subject standard deviation. The delta-method^47^ was then used to obtain approximate standard errors of $PCT_k$ and $ES_k$ along with 95% confidence limits. In addition, as measure of programmatic risk, we also used mixed-model regression, this time to estimate *P*_20_, the proportion of study subjects that would be expected to have a 20% or greater loss at the first postflight session. It is not feasible to use the raw data directly to calculate this proportion because of the relatively few numbers of subjects and the variability of the preflight baseline measurements. Instead, we used another version of the mixed-model, but applied to the preflight and first postflight data along with the possible inclusion of (a) a postflight random interaction (the variability of the slopes in Supplemental Figure 4 where the slopes are considered “random” because they vary unpredictably between subjects) as well as (b) the inclusion of body weight as a covariate. Depending on the endpoint, neither, either, or both of (a) and (b) were used in the model as decided by an automated process based on model-fit criteria. The delta method was also used to obtain a standard error and 95% confidence limits for *P*_20_. All model fitting was done using Stata Statistical Software. Given the many predictor and response variables, we elected to portray groups of associations in a holistic way as opposed to identifying which specific predictors appear to affect a specific response. Association between change in endpoint variables and inflight predictor variables was quantified in terms of the rank-based Somers’ D^10^ to allow for non-linearity and control the effect of outliers.

### Reporting summary

Further information on research design is available in the Nature Portfolio Reporting Summary linked to this article.

## Supplementary information

Supplemental Material

Reporting Summary Checklist

## Data Availability

Data from this study may be obtained through a data request to the NASA Life Science Data Archive (https://lsda.jsc.nasa.gov/Request/dataRequest).
