# Supplementary material for: Effects of exercise countermeasures on multisystem function in long duration spaceflight astronauts
Source: NPJ Microgravity. 2023 Feb 3;9:11. doi: 10.1038/s41526-023-00256-5 (PMC9898566; doi:10.1038/s41526-023-00256-5)
Supplement: Supplementary file 1 — Supplemental Material [file 41526_2023_256_MOESM1_ESM.pdf]

SUPPLEMENTAL MATERIAL

Effects of Exercise Countermeasures on Multisystem Function in Long Duration Spaceflight Astronauts

Table of Contents

1. **Supplemental Figure 1.** The Second Generation Treadmill (T2) on ISS
2. **Supplemental Figure 2.** Cycle Ergometer with Vibration Isolation System (CEVIS) on ISS
3. **Supplemental Figure 3.** Advanced Resistive Exercise Device (ARED) on ISS
4. **Supplemental Figure 4.** Inter-individual Heterogeneity in Response from Pre- to Post-flight
5. **Supplemental Table 1.** In-flight Daily Caloric and Macronutrient Intake
6. **Supplemental Table 2.** Data Collection Schedule and Astronaut Participation
7. **Supplemental Table 3.** Pre and Postflight Bone Mineral Density and Content
8. **Supplemental Table 4.** Pre and Postflight Muscle Strength and Size
9. **Supplemental Table 5.** Pre and Postflight Cardiorespiratory Fitness
10. **Supplemental Table 6.** Pre and Postflight Body Composition

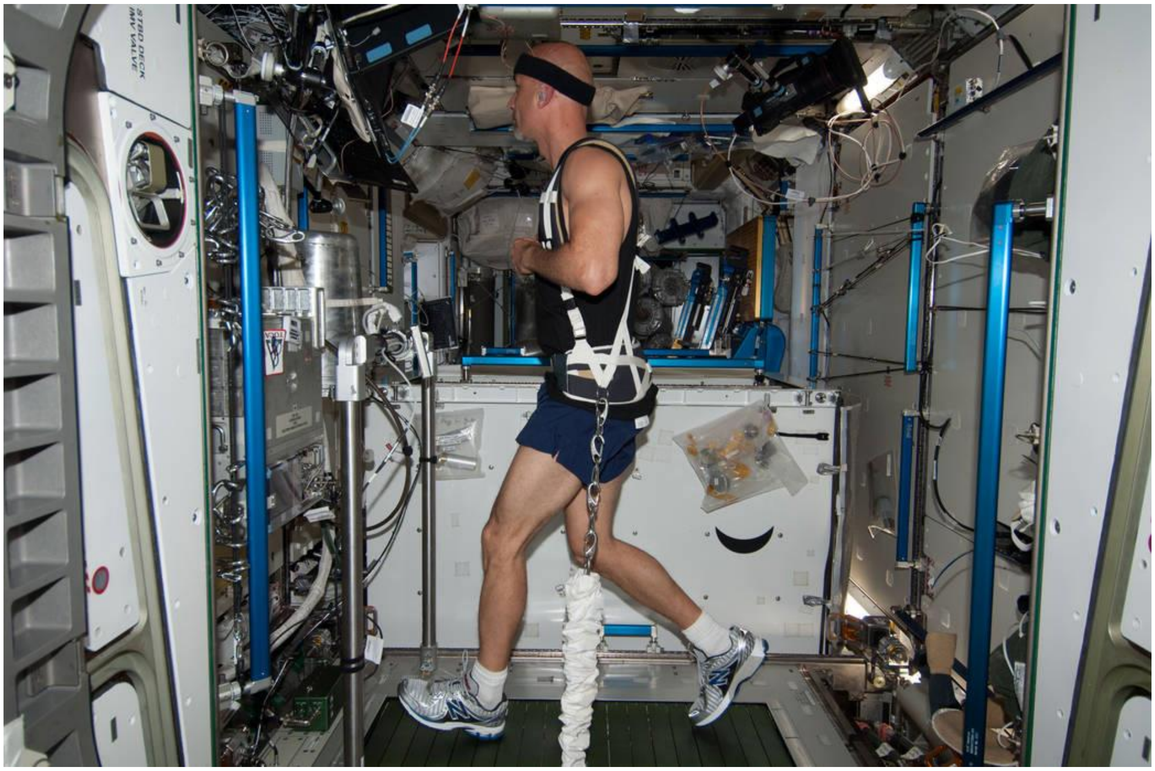

Supplemental Figure 1. The Second Generation Treadmill (T2) on ISS. NASA Image: ISS036-E-005384; https://www.nasa.gov/multimedia/guidelines/index.html

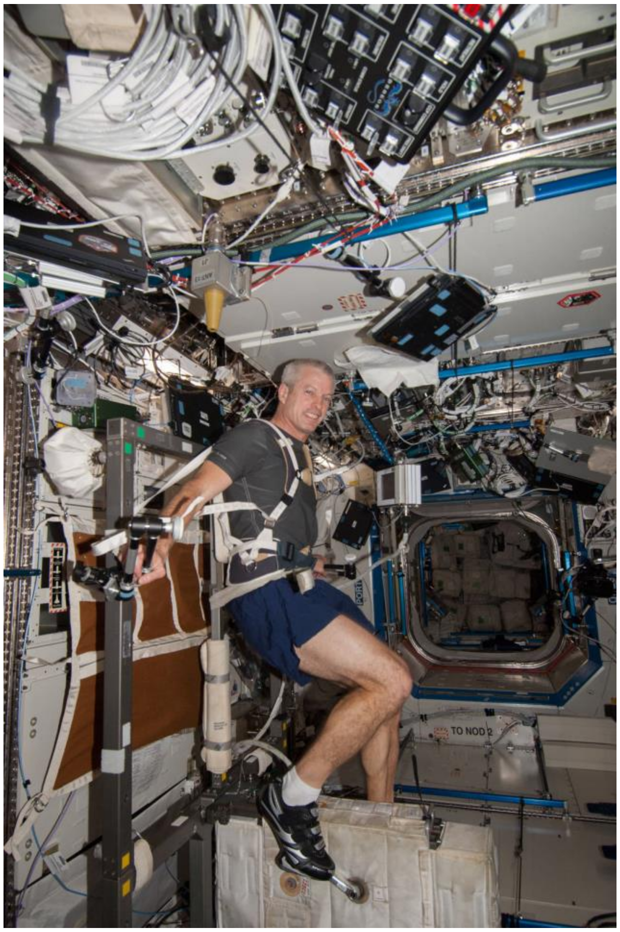

Supplemental Figure 2. Cycle Ergometer with Vibration Isolation System (CEVIS) on ISS. NASA Image: SS039-E-014696; https://www.nasa.gov/multimedia/guidelines/index.html

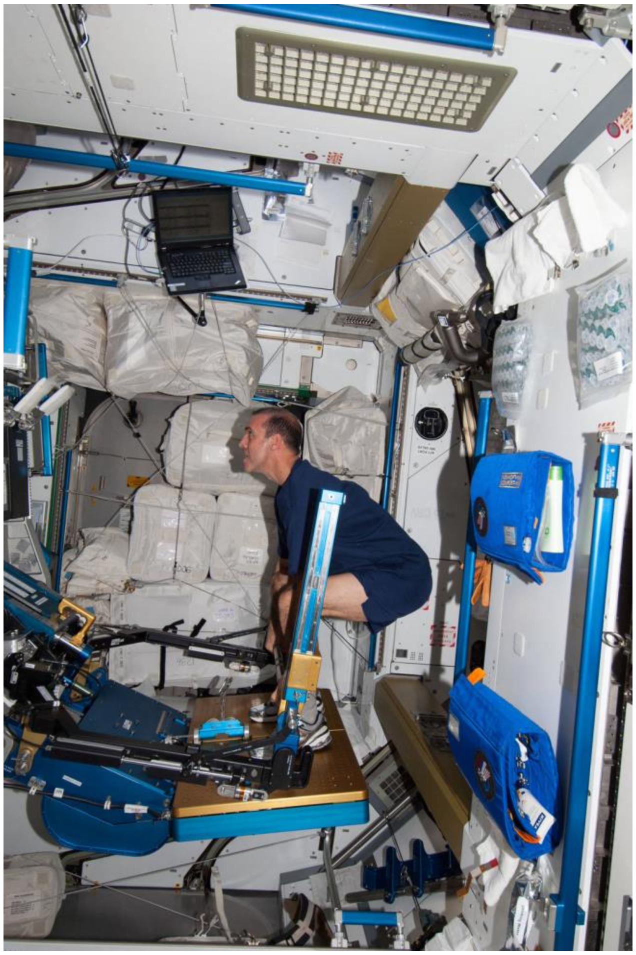

Supplemental Figure 3. Advanced Resistive Exercise Device (ARED) on ISS. NASA Image: ISS038-E-000281; https://www.nasa.gov/multimedia/guidelines/index.html

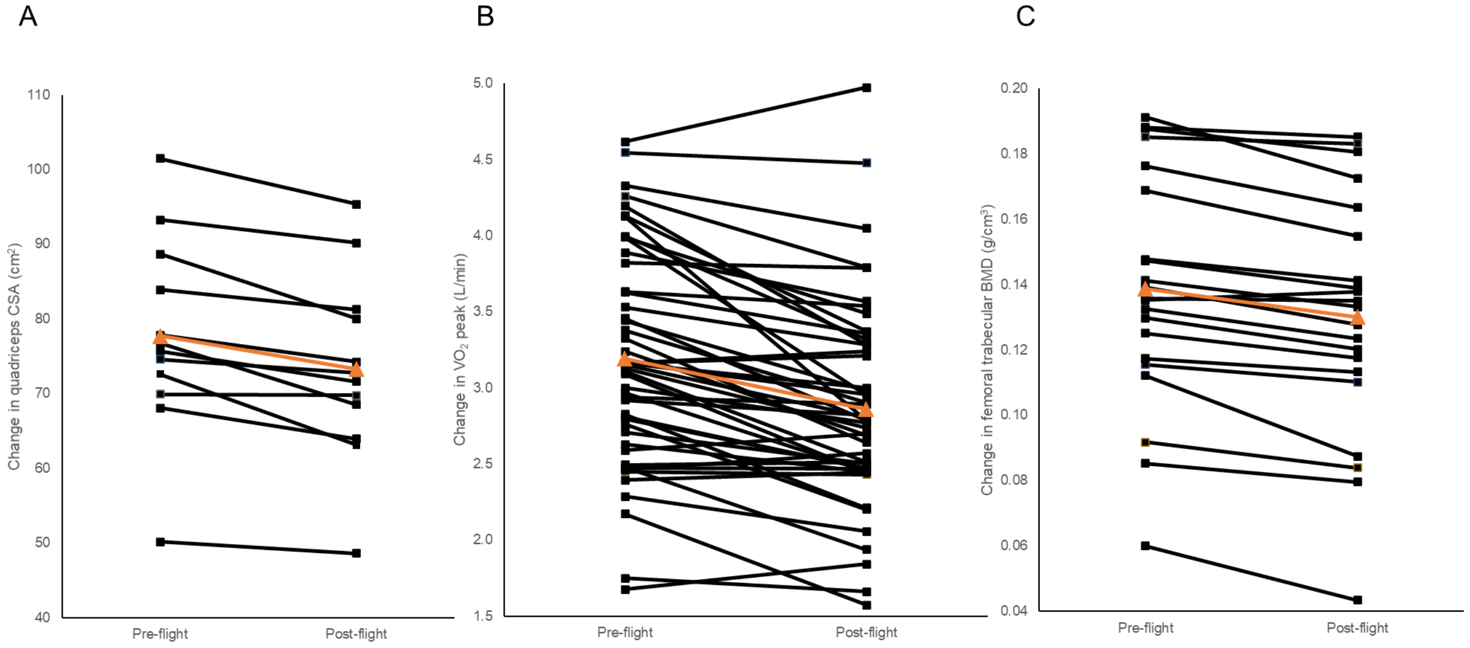

Supplemental Figure 4. Inter-individual Heterogeneity in Response from Pre- to Post-flight. (A) Individual responses for quadriceps size. (B) Individual responses for VO_2peak. (C) Individual responses for bone content in trabecula femur. Abbreviations: CSA, cross sectional area; VO_2peak, peak oxygen consumption; BMD, bone mineral density.

Supplemental Table 1. In-flight Daily Caloric and Macronutrient Intake.

	Total	Relative to body weight, kg
Total calories (kcal)	2296 ± 449	29 ± 5
Protein (g)	112 ± 24	1.4 ± 0.3

Values are expressed as means ± SD.

Supplemental Table 2. Data Collection Schedule and Astronaut Participation.

System	N	Days before launch (preflight)	Days after Return (postflight)	Individual endpoints
**Muscle strength, power, and size**				
Isometric and ballistic leg press and bench press	17	30, 85	1, 7, 30	Leg press and bench press: force, power, total work
One-repetition maximum leg press and bench press	38	60, 90	1, 7	Leg press and bench press 1-RM
Cross sectional area (MRI)	12	40	1	Quadriceps, hamstring, calf
**Cardiorespiratory fitness**	21	28, 90	2, 7, 30	VO_2peak, ventilatory threshold, ventilation
**Body composition (DXA)**				
Muscle and fat mass	44	90	7	Total mass, fat mass, lean mass
Bone	44	90	7	Total hip, trochanter, femoral neck, L1-L4:
**Bone volume (QCT)**	12	90	7	Cortical and trabecular total femur, trochanter, femoral neck

Abbreviations: RM: repetition maximum; MRI, magnetic resonance imaging; DXA, Dual Energy X-ray Absorptiometry; QCT, quantitative computed tomography; L, lumbar.

Supplemental Table 3. Pre and Postflight Bone Mineral Density and Content.

	Preflight	N	Postflight	N
DXA				
Total hip (g/cm^2)	1.04±0.10	44	1.00±0.11	44
Trochanter (g/cm^2)	0.77±0.09	44	0.75±0.10	44
Femoral neck (g/cm^2)	0.86±0.10	44	0.84±0.10	44
L1-L4	1.09±0.13	44	1.06±0.13	44
QCT Trabecular				
Total Femur (g/cm^3)	0.143±0.03	12	0.137±0.03	12
Trochanteric (g/cm^3)	0.144±0.03	12	0.138±0.03	12
Femoral neck (g/cm^3)	0.133±0.04	12	0.122±0.04	12
QCT Cortical				
Total Femur (g/cm^3)	0.519±0.05	12	0.513±0.05	12
Trochanteric (g/cm^3)	0.524±0.05	12	0.516±0.05	12
Femoral neck (g/cm^3)	0.554±0.07	12	0.552±0.07	12

Values are means ± SD. Abbreviations: DXA, dual-energy x-ray absorptiometry; QCT, quantitative computed tomography

Supplemental Table 4. Pre and Postflight Muscle Strength and Size.

	Preflight	N	Postflight	N
Leg Press Force (N)	2027±500	15	1912±486	17
Leg Press Total Work (W)	8338±2198	14	7572±1985	17
Leg Press Max Power (W)	1829±405	14	1647±370	17
Leg Press 1-RM (lb)	639±154	38	614±144	39
Bench Press Force (N)	966±278	16	945±247	17
Bench Press Total Work (W)	3693±1147	16	3598±1169	17
Bench Press Max Power (W)	849±244	16	838±280	17
Bench Press 1-RM (lb)	181±46	38	186±49	37
Hamstring CSA (cm^2)	33.8±3.5	12	31.1±3.7	12
Quad CSA (cm^2)	77.7±13.2	12	73.3±12.5	12
Calf CSA (cm^2)	17.7±4.1	12	15.6	12

Values are mean ± SD. Abbreviations: RM, repetition maximum; CSA, cross sectional area.

Supplemental Table 5. Pre and Postflight Cardiorespiratory Fitness.

	Preflight	N	Postflight	N
VO_2peak (L/min)	3.16±0.05	26	2.93±0.06	21
Ventilation (ml)	135.8±2.8	26	130.0±3.5	21
Ventilatory Threshold (L/min)	2.84±0.07	22	2.88±0.06	13

Values are mean ± SD. Abbreviations: VO_2peak; peak oxygen consumption

Supplemental Table 6. Pre and Postflight Body Composition.

	Preflight	N	Postflight	N
Total Mass (kg)	78.3±9.8	44	78.0±10.2	44
Fat (kg)	19.2±4.6	44	18.7±4.9	44
Lean (kg)	56.2±7.8	44	56.5±8.3	44

Values are means ± SD.
