# Supplementary material for: Effects of exercise countermeasures on multisystem function in long duration spaceflight astronauts
Source: NPJ Microgravity. 2023 Feb 3;9:11. doi: 10.1038/s41526-023-00256-5 (PMC9898566; doi:10.1038/s41526-023-00256-5)
Supplement: Supplementary file 2 — Reporting Summary Checklist [file 41526_2023_256_MOESM2_ESM.pdf]

nature portfolio

Corresponding author(s): Jessica Scott

Last updated by author(s): Oct 11, 2022

Reporting Summary

Nature Portfolio wishes to improve the reproducibility of the work that we publish. This form provides structure for consistency and transparency in reporting. For further information on Nature Portfolio policies, see our Editorial Policies and the Editorial Policy Checklist.

Statistics

For all statistical analyses, confirm that the following items are present in the figure legend, table legend, main text, or Methods section.

n/a	Confirmed	
☐	☒	The exact sample size (n) for each experimental group/condition, given as a discrete number and unit of measurement
☐	☒	A statement on whether measurements were taken from distinct samples or whether the same sample was measured repeatedly
☐	☒	The statistical test(s) used AND whether they are one- or two-sided<br>*Only common tests should be described solely by name; describe more complex techniques in the Methods section.*
☒	☐	A description of all covariates tested
☐	☒	A description of any assumptions or corrections, such as tests of normality and adjustment for multiple comparisons
☐	☒	A full description of the statistical parameters including central tendency (e.g. means) or other basic estimates (e.g. regression coefficient) AND variation (e.g. standard deviation) or associated estimates of uncertainty (e.g. confidence intervals)
☐	☒	For null hypothesis testing, the test statistic (e.g. F, t, r) with confidence intervals, effect sizes, degrees of freedom and P value noted<br>*Give P values as exact values whenever suitable.*
☒	☐	For Bayesian analysis, information on the choice of priors and Markov chain Monte Carlo settings
☒	☐	For hierarchical and complex designs, identification of the appropriate level for tests and full reporting of outcomes
☐	☒	Estimates of effect sizes (e.g. Cohen's d, Pearson's r), indicating how they were calculated

Our web collection on statistics for biologists contains articles on many of the points above.

Software and code

Policy information about availability of computer code

Data collection	DXA: Hologic Discovery; Hologic Inc., Waltham, MA, USA<br>QCT General Electric Advantage QXi<br>Isokinetic: Biodex Medical Systems, Shirley, NY<br>MRI: Image-J (National Institutes of Health, Bethesda, MD, USA, version 1.42)<br>Nutrition: Nutrition Data System for Research software versions 2007, 2010, 2012, and 2014, developed by the Nutrition Coordinating Center, University of Minnesota<br>VO2: Portable Pulmonary Function System (PPFS)
Data analysis	Statistics: Stata Statistical Software (V15.1)

For manuscripts utilizing custom algorithms or software that are central to the research but not yet described in published literature, software must be made available to editors and reviewers. We strongly encourage code deposition in a community repository (e.g. GitHub). See the Nature Portfolio guidelines for submitting code & software for further information.

Data

Policy information about availability of data

All manuscripts must include a data availability statement. This statement should provide the following information, where applicable:

- Accession codes, unique identifiers, or web links for publicly available datasets
- A description of any restrictions on data availability
- For clinical datasets or third party data, please ensure that the statement adheres to our policy

Data from this study may be obtained through a data request to the NASA Life Science Data Archive (https://lsda.jsc.nasa.gov/Request/dataRequest)

Human research participants

Policy information about studies involving human research participants and Sex and Gender in Research.

Reporting on sex and gender	Forty-six astronauts (37 males, 9 females) were included. Sex was included in evaluation of factors associated with multisystem responses to spaceflight.
Population characteristics	Forty-six astronauts (37 males, 9 females; age: 46.8 ± 6.1 years, height: 176 ± 7.1 cm, weight: 79.2 ± 9.9 kg [mean ± SD]) were included.
Recruitment	All astronauts completed standard preflight medical screening, received clearance from flight surgeons, and provided written informed consent before participating in the study. All astronauts included in this paper performed the standard medically required physiologic tests involving muscle strength, aerobic fitness, and bone health; a subset performed additional experimental tests of muscle strength and size, aerobic fitness, and bone health.
Ethics oversight	This study was approved by the Institutional Review Board at NASA Johnson Space Center (JSC, Houston, TX), the Japan Aerospace Exploration Agency (JAXA) Institutional Review Board, the European Space Agency (ESA) Medical Board, and the Human Research Multilateral Review Board.

Note that full information on the approval of the study protocol must also be provided in the manuscript.

Field-specific reporting

Please select the one below that is the best fit for your research. If you are not sure, read the appropriate sections before making your selection.

☒ Life sciences ☐ Behavioural & social sciences ☐ Ecological, evolutionary & environmental sciences

For a reference copy of the document with all sections, see nature.com/documents/nr-reporting-summary-flat.pdf

Life sciences study design

All studies must disclose on these points even when the disclosure is negative.

Sample size	Sample size was determined by the NASA Human Research Program based on available data from ISS astronauts.
Data exclusions	No data were excluded.
Replication	There were no attempts at replication due to the nature of the investigation.
Randomization	All National Aeronautics and Space Administration (NASA), Canadian Space Agency (CSA), European Space Agency (ESA), and Japan Aerospace Exploration Agency (JAXA) astronauts assigned to ISS flight were eligible to participate in this investigation. There was no randomization.
Blinding	Participants and investigators were not blinded due to the nature of the investigation.

Reporting for specific materials, systems and methods

We require information from authors about some types of materials, experimental systems and methods used in many studies. Here, indicate whether each material, system or method listed is relevant to your study. If you are not sure if a list item applies to your research, read the appropriate section before selecting a response.

Materials & experimental systems

n/a	Involved in the study
☒	☐ Antibodies
☒	☐ Eukaryotic cell lines
☒	☐ Palaeontology and archaeology
☒	☐ Animals and other organisms
☒	☐ Clinical data
☒	☐ Dual use research of concern

Methods

n/a	Involved in the study
☒	☐ ChIP-seq
☒	☐ Flow cytometry
☒	☐ MRI-based neuroimaging
